# Hybrid Technique for Abdominal Wall Hernia Repair: Description and Early Results

**DOI:** 10.7759/cureus.62882

**Published:** 2024-06-22

**Authors:** Mohit Bhatia, Sharmila Vijayan, Doaa Al-Maliki, Elia Azir, Shamsi El-Hasanii

**Affiliations:** 1 Surgery, Princess Royal University Hospital, King's College Hospital NHS Foundation Trust, London, GBR; 2 General Surgery, Princess Royal University Hospital, King's College Hospital NHS Foundation Trust, London, GBR; 3 Upper Gastrointestinal Surgery, Princess Royal University Hospital, King's College Hospital NHS Foundation Trust, London, GBR

**Keywords:** hybrid repair, minimally invasive laparoscopy, laparoscopy, laparoscopic ventral hernia repair, ventral hernia

## Abstract

Objective

Ventral hernia repair is a widely practiced surgical procedure worldwide. The objective of this paper is to evaluate and analyze the results of a hybrid approach for treating ventral hernias.

Methods

All patients with clinically and radiologically proven ventral hernia underwent hybrid laparoscopic ventral hernia repair at Princess Royal University Hospital, London, United Kingdom using a retrospective approach with the same surgical team. Large defects >10 cm, inguinal hernia, para-stomal hernia, incarcerated patients, and spigelian hernia were excluded. We utilized the laparoscopic approach for the dissection and isolation of the sac and used the port site for the delivery of mesh into the abdominal cavity.

Results

Our study comprises 67 patients, with 39 males (58.2%) and 28 females (41.8%). The median age in our study group was 41 years (range: 18-65 years). The median BMI was 38 kg/m^2 ^(range: 24-52 kg/m^2^). The majority of the cases were umbilical or paraumbilical hernias (n = 46). The median defect size in our study was 5.4 cm (range: 2-10 cm). The median operative time was 67 minutes. We have not encountered any recurrences in this group.

Conclusion

This hybrid approach combines the advantages of both the open and laparoscopic approaches.

## Introduction

Abdominal wall hernia repair is a surgical procedure aimed at addressing abdominal wall weaknesses and defects. The main reason for developing ventral wall hernias is weakness in the fasciomuscular layer of the abdominal wall [1]. With the wide acceptance of laparoscopic techniques, this approach for treating ventral hernia has also gained popularity overall [2]. In 1993, Leblanc and Booth started the concept of laparoscopic ventral hernia repair (LVH) [3].

Numerous studies have demonstrated the advantages of the laparoscopic approach in managing ventral hernia by reducing postoperative pain and hospital stay [4]. However, some studies have questioned the efficacy of the laparoscopic approach in relation to seroma formation and compromised cosmetic results. As the hernia sac is left in situ, this will predispose to seroma formation and compromise cosmetic results. It is also suggested that this approach has limitations, especially when the defect size is large, and that the prolonged operative times further dent the prospects of the laparoscopic approach [5].

Open hernia repair, on the other hand, offers easy access for the placement of mesh and enables complete resection of the hernia sac, thereby avoiding the risk of bowel injury and reducing postoperative bulge and seroma formation [6]. Due to the scarcity of evidence to suggest a superior technique for managing ventral hernia, some authors presented modifications in the surgical approach to get better results by incorporating both techniques. They suggested that closure of the sheath defect, along with the laparoscopic approach, yields low recurrence rates and is associated with a low incidence of chronic pain [7].

Our aim is to share the technique that we believe has advantages and can be used in the majority of abdominal wall hernias, which reflects modifications in the surgical technique that may be of benefit to the surgical community worldwide. We call the technique “hybrid repair,” which we believe combines the benefits of the open and laparoscopic approaches together.

## Materials and methods

All our patients (n = 67) who underwent a hybrid hernia repair technique at Princess Royal University Hospital, London, United Kingdom were included in this retrospective study. This study included patients enrolled from March 2020 to March 2023. All these patients were evaluated and operated on by the same surgical team.

Inclusion criteria

All patients above the age of 18 with clinically and radiologically proven ventral hernia (umbilical/paraumbilical, spigelian, epigastric, or incisional hernia) were included in this study.

Exclusion criteria

Patients with a defect size >10 cm and a clinical diagnosis of inguinal hernia, lumbar hernia, incarcerated hernia, and parastomal hernia were excluded from this study.

All patients who met the inclusion criteria and were deemed fit for general anesthesia were evaluated in this study. All the patients were clinically assessed; routine blood investigations, ultrasound in all cases, and CT scans (for incisional hernia) were performed. Informed consent and anesthetic evaluation were obtained before any surgical intervention. All patients were given IV antibiotics (co-amoxiclav) at the time of induction. General anesthesia was used in all cases.

Surgical technique

**Figure 1:**
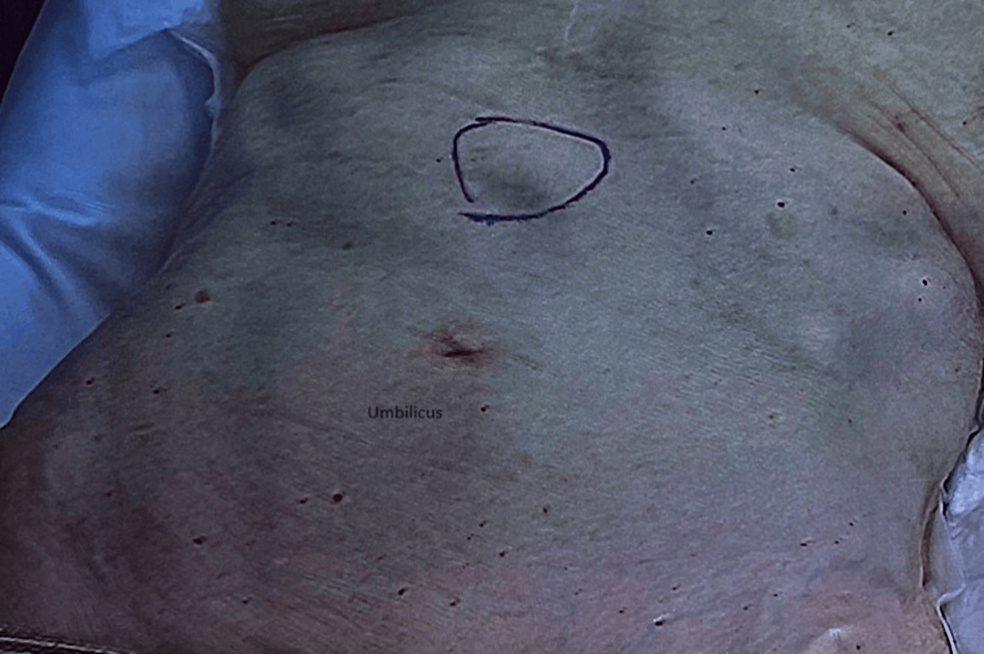
Ventral hernia marked preoperatively

**Figure 2:**
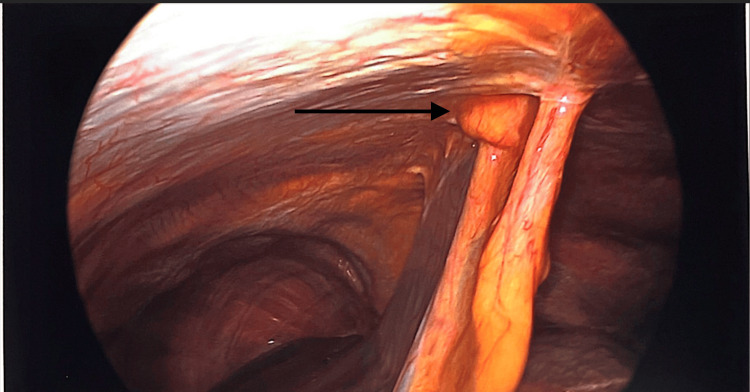
Hernia defect on the right side of the falciform ligament

**Figure 3:**
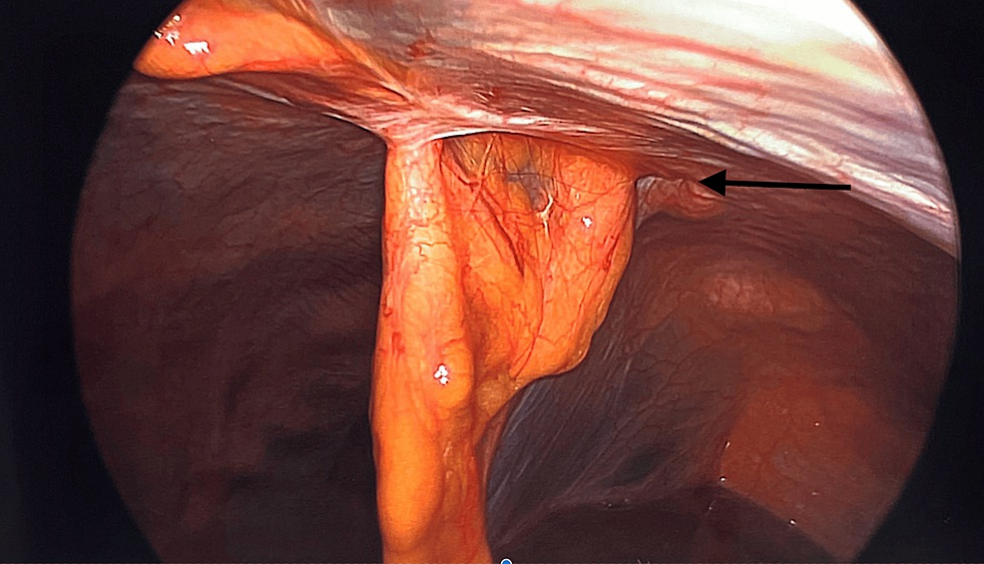
Hernia defect from the left side of the falciform ligament

**Figure 4:**
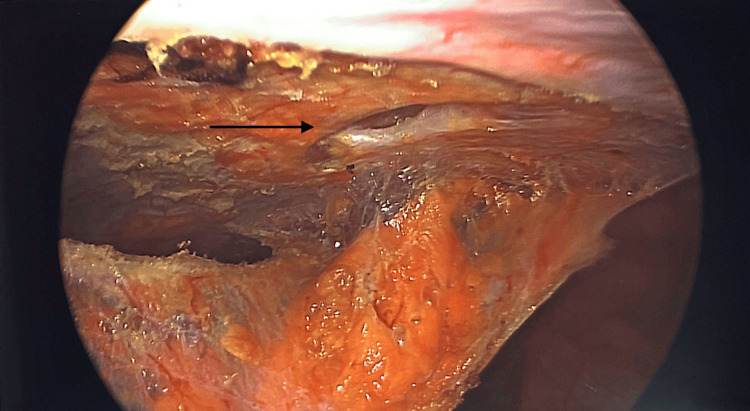
Defect clearly seen after the dissection

**Figure 5:**
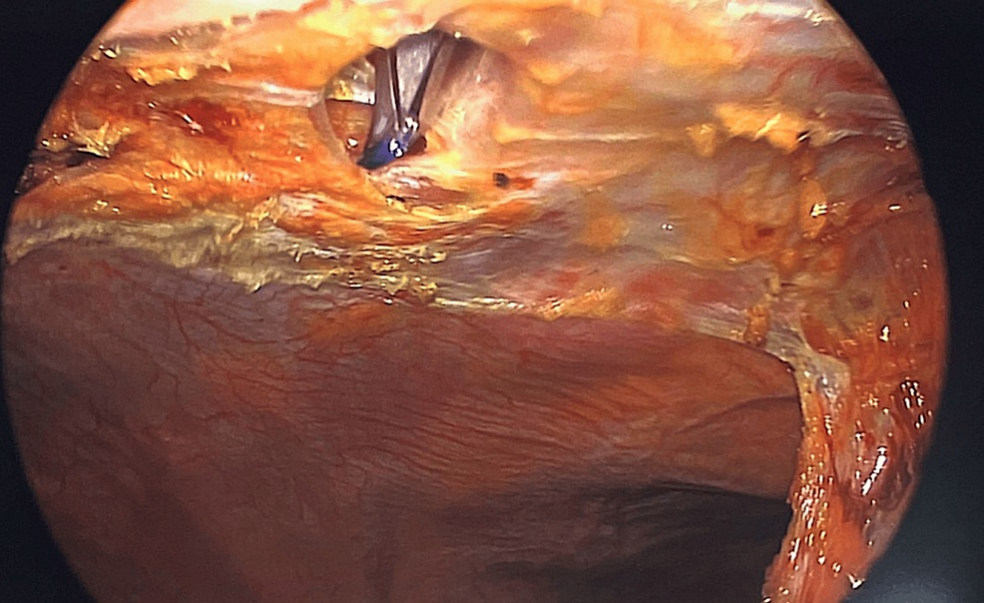
Passing the port through the defect

Patients were positioned in the supine position. Pneumoperitoneum was induced via a Veress needle. Five-millimeter ports were inserted and adjusted according to the location of the hernia to provide the triangulation required to perform surgery. The hernia was identified, and the contents were clearly reduced, depending on the size of the sac and whether it is reduced and excised laparoscopically or left in situ to be excised when we make the anterior abdominal wall incision. The preperitoneal fat and sometimes the falciform ligament around the defect will be dissected to allow clear demarcation of the main defect and to identify other occult defects if they are encountered (Figures 1-5). At this stage, an anterior abdominal wall incision was placed over the well-identifiable edge of the defect. This incision will be relatively small, only enough to make dissection to excise the sac if it was still in situ, and equally important just to clearly identify the edges of the defect.

Defects were closed by an interrupted polydioxanone suture (PDS), only leaving a gap to insert a 12-mm port through the defect. Pneumoperitoneum was induced again after measuring the defect in both directions. The appropriate size of Parietex circular mesh (usually 9 cm or 12 cm) was introduced through the port into the abdominal cavity with a stitch in the center to help eventually pull the mesh in the center of the defect against the abdominal wall before fixation.

The center of the mesh positioned in the middle of the defect pneumoperitoneum was reduced, and the port was removed. We continued to close the abdominal wall defect anteriorly using the interrupted PDS, and then another layer of continuous PDS will be used as well. Pneumoperitoneum was induced again, but at 8 mmHg, the mesh was pulled by the suture up against the abdominal wall in the center of the closed defect. At this stage, the mesh was spread widely and fixed with an absorbable tacker.

**Figure 6:**
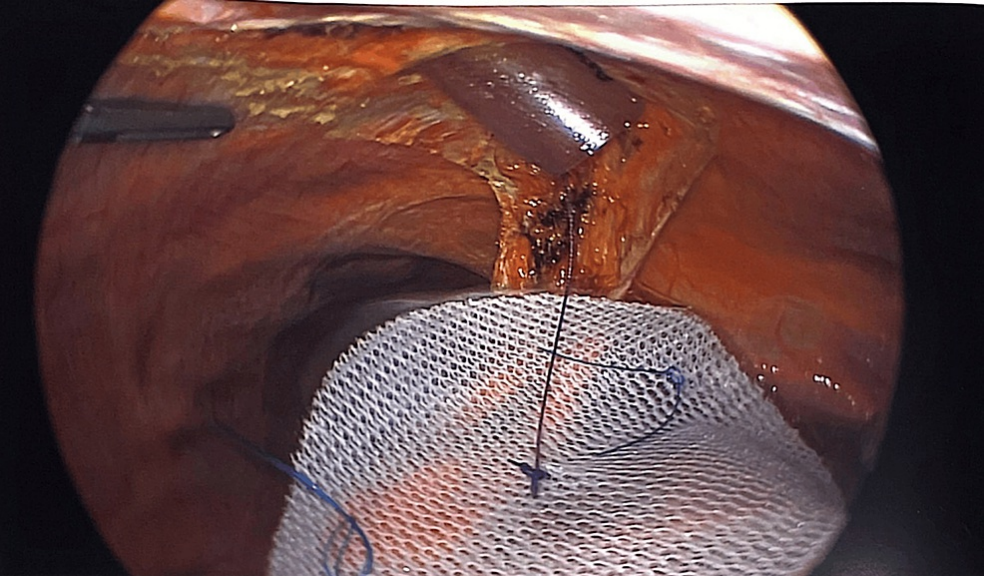
Delivery of mesh into the peritoneal cavity

**Figure 7:**
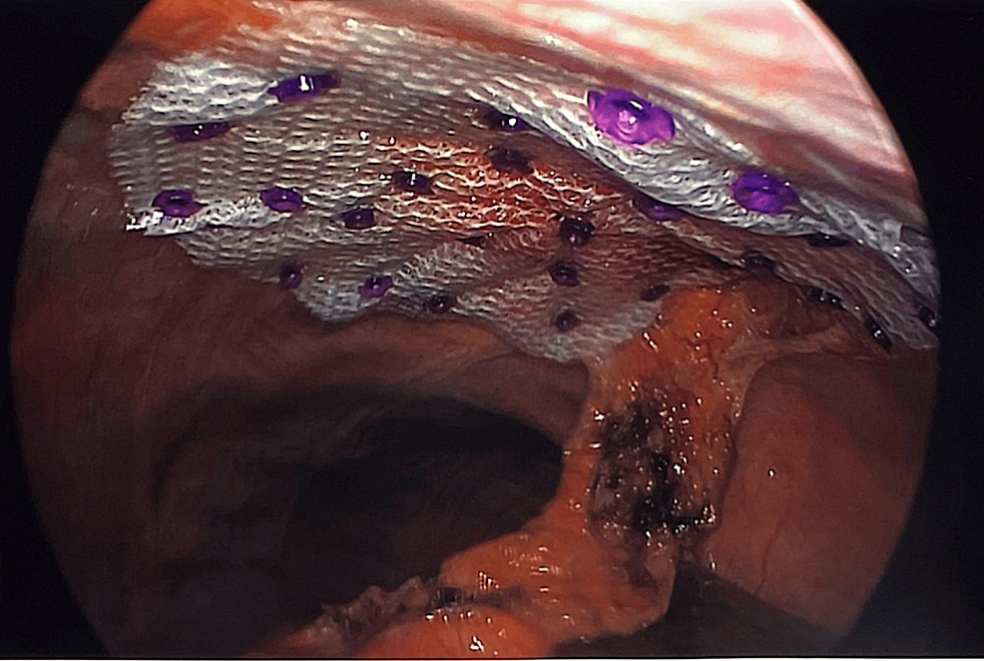
Mesh placed and fixed with tackers to ensure complete coverage of the defect

**Figure 8:**
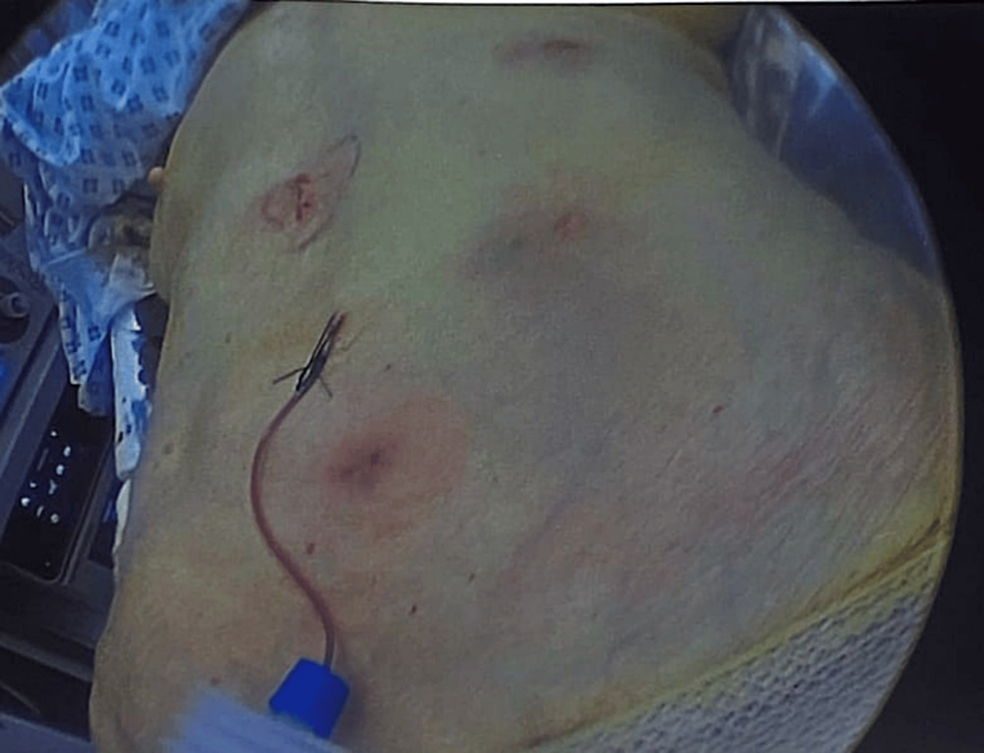
Postoperative wound sites with a small Medivac drain

Port wounds are closed in layers, and there is fixed subcutaneous tissue. A drain will be left in the anterior abdominal wall incision that was made over the hernia. We closed the skin with absorbable stitches (Figures 6-8).

All patients were mobilized the same day, mostly within two hours after the surgery. All patients except one were discharged home the same day and followed up in the clinic four to six weeks postoperatively as face-to-face assessments and six monthly telephonic assessments. Any form of intraoperative or postoperative complications was assessed.

## Results

**Figure 9:**
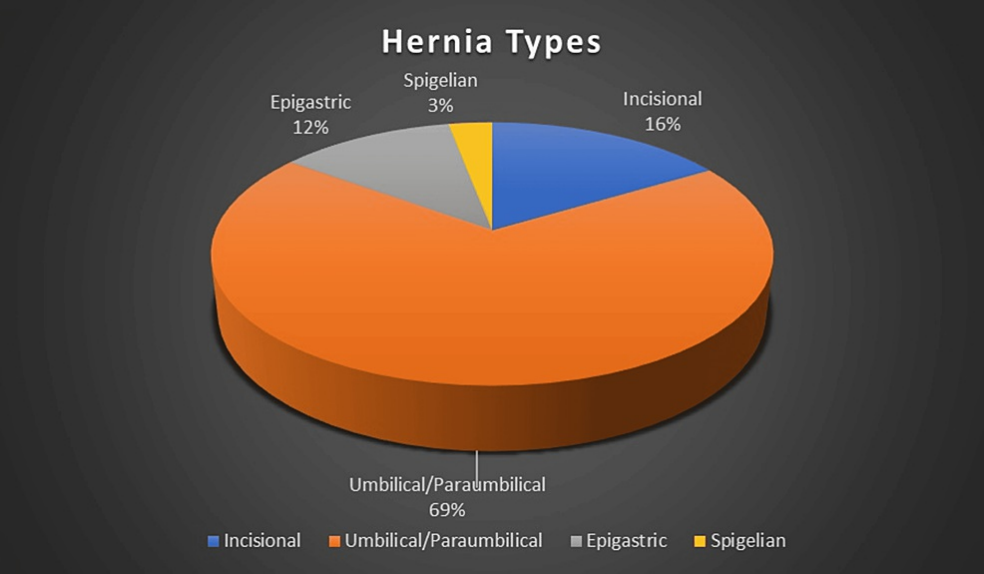
Case distribution in our study

In our sample size of 67, 39 patients were males (n = 58.2%) and 28 patients were females (n = 28; 41.8%). The median age in our study group was 41 years (range: 18-65 years). The median BMI was 38 kg/m^2^ (range: 24-52 kg/m^2^). Total cases included (n = 67) umbilical/paraumbilical hernia (n = 46), incisional (n = 11), epigastric (n = 8), and spigelian (n = 2). This included three recurrent cases (one incisional and two umbilical hernias) in which the previous mesh was removed (Figure 9).

The median defect size in our study was 5.4 cm (range: 2-10 cm). The median operative time was 67 minutes (range: 45-100 minutes). We used AbsorbaTack 5 mm by Covidien (Dublin, Ireland) in all cases. In all our patients, mobilization was encouraged two hours after the surgery, and a return to normal activities was achieved four weeks postoperatively. Patients were followed up in the clinic six weeks following surgery, then virtually every six months in 43 patients (64.2%), completing a six-month follow-up. We did not encounter any intraoperative or postoperative complications, except for one patient who developed a wound infection and required a return to the theater for wound debridement and washout. We have followed up on these cases and will be having clinical assessments to record postoperative recovery.

Patients receiving planned hybrid hernia repair is an important aspect of safeguarding postoperative recovery, and it helps in ensuring patient safety and satisfaction with the results.

## Discussion

Abdominal wall hernias are common in surgical practice, and both the laparoscopic and open repair approaches have been widely used [8]. In our hands, the hybrid approach using the open and laparoscopic approaches combined the benefits of both techniques and decreased the complications. The laparoscopic approach will define the contents of the hernia, expose occult defects that will be repaired by the mesh as well, and make the reduction of the contents safe and under direct vision. The mesh was placed laparoscopically, giving strength to the repair by fixing the mesh to the posterior aspect of the fasciomuscular defect.

With our adopted hybrid surgical technique, we incorporated both the open and laparoscopic approaches, which enabled us to achieve satisfactory results without encountering complications. The laparoscopic approach will identify occult and multiple hernia defects and is more suited for high-BMI patients. Contrary to the open conventional techniques, which may be associated with high morbidities and increased recurrence rates [9], our study shows results comparable to those of another study that utilized the hybrid method of ventral hernia repair [10].

We place mesh laparoscopically in our hybrid technique with the closure of the defect using the open approach; it is considered a safe approach, especially in complex ventral hernias, multiple defects, and high BMI patients [11]. It is widely accepted that the recurrence of hernia is multi-actorial; however, modifications in the surgical approach, especially by experienced surgical professionals, can help in negating the recurrence risk factors such as large hernia size, high BMI, difficult access cases, and adequate strengthening of the defect with the mesh [3].

Bernardi et al. [12] concluded that closing the defect during LVH results in better cosmesis and better outcomes. In our adoption of this technique, we have been using the laparoscopic approach for fixing the mesh, which ultimately decreases the chances of mesh migration and hence the low recurrence rates. A randomized study showed the rate of seroma formation in 45% of patients treated with a hybrid surgical approach compared to 30% with a laparoscopic approach [13]. Contrary to this, in our experience, we did not encounter any patients with seroma formation. We feel that with our technique, there is minimal handling as the aim of the anterior abdominal wall incision is only to display the edges of the abdominal wall defect without raising the skin and subcutaneous flaps widely, and we routinely use a drain in the subcutaneous layer. However, the literature suggests a seroma formation incidence of up to 16% associated with the closure of the defects [14].

Our study has the limitation of having a small sample size and experience from a single institution, and this may not represent a general opinion. However, with this study, we tried to emphasize that the hybrid technique for the repair of ventral hernia in experienced hands can help reduce the morbidities associated with hernia repair.

## Conclusions

This hybrid approach combines the advantages of both the open and laparoscopic approaches. The defect will be closed, a mesh will be placed, and the sac will be excised. There is less risk of seroma and hematoma, and the majority are day cases or overnight stays in the hospital. Early results are encouraging, with good patient satisfaction.
